# The functions and clinical application potential of exosomes derived from adipose mesenchymal stem cells: a comprehensive review

**DOI:** 10.1186/s13287-019-1358-y

**Published:** 2019-08-07

**Authors:** Pengyu Hong, Hao Yang, Yue Wu, Kun Li, Zhangui Tang

**Affiliations:** Department of Oral & Maxillofacial Surgery, Xiangya Stomatological Hospital & School of Stomatology, Central South University, Changsha, 410008 Hunan China

**Keywords:** Adipose-derived stem cells, Exosomes, Function, Clinical application

## Abstract

Exosomes are extracellular membranous nanovesicles that mediate local and systemic intercellular communication by transporting proteins or nucleic acids (DNA and RNA) into target cells, thus altering the behaviors of recipient cells. Recent studies have revealed that these vesicles play a critical role in many biological functions, such as cell proliferation, immune regulation, nerve regeneration, and cancer. Adipose-derived stem cells (ADSCs) are now considered a multipotent and abundant tool in the field of cell therapy and regenerative medicine. ADSCs can produce and secrete many exosomes, which inherit multiple functions of cells. Therefore, in this review, we will introduce the characteristics of exosomes derived from ADSCs (ADSC-Exos), describe their functions in different biological processes, summarize the latest research achievements, describe their limitations in cell-free therapy, and provide further insights into their clinical application potential for the treatment of certain diseases.

## Introduction

Mesenchymal stem cells (MSCs) have gradually become one of the most promising cell therapy tools because of their relatively simple procedure for cell isolation, self-renewal and expansion potential, low immunogenicity, multipotency, and secretion of mediators that support tissue renovation or substitution [1]. Moreover, ADSCs, which are derived from stromal-vascular fragments of adipose tissue, have also shown credible and reliable clinical application value [2]. They are capable of differentiating into diverse cell lineages and secreting high levels of proteins that function in immunoregulation, angiogenesis, revascularization, cutaneous wound healing, and tissue regeneration [3, 4]. In addition to secreted proteins, cells can also release exosomes, which are defined as small extracellular vesicles (EVs) with a multivesicular endosomal origin [5]. ADSC-Exos, whether in normoxic or hypoxic environments, have been successfully isolated in vitro [6, 7]. NanoSight analysis indicated that the size of ADSC-Exos and hypoxia-preconditioned ADSC-Exos ranged from 20 to 300 nm with a mean size of 90 nm [6]. Accumulating evidence has indicated and verified that exosomes derived from stem cells transfer proteins [8], mRNAs/microRNAs (miRNAs) [9, 10], or even DNA molecules [11] from cell to cell via paracrine or endocrine signaling [12]. Therefore, various studies have demonstrated that exosomes are novel frontiers of intercellular communication regulating the biological behaviors of cells, such as angiogenesis [13], immune modulation [14], proliferation, and migration [15]. Owing to their multiple functions, exosomes have shown strong diagnostic and therapeutic potential in many clinical diseases, especially for the treatment of tumors [10, 16]. In addition, exosomes have been utilized for targeted drug delivery and as gene carriers for regenerative medicine [17].

Accordingly, ADSC-Exos may play pivotal roles in tissue engineering and regenerative therapies [2]. In this review, we will illustrate the characteristics of ADSC-Exos and highlight their functions and clinical application potential with a focus on the most recent literature to provide and summarize the current valuable knowledge in this area.

## Characteristics of exosomes

EVs, which are referred to as membrane-packed vesicles, were recognized by Pan et al. during the maturation of reticulocytes in 1983 [14]. Meanwhile, Trams et al. found that various cells excreted vesicles with 5′-nucleotidase activity and introduced them with the term “exosomes” [18], which comprise one of the main subclasses of EVs and have an endosomal origin [19]. Exosomes are 40–100-nm vesicles, and their buoyant density in sucrose ranges from 1.10 to 1.21 g/ml [8]. Studies have shown that exosomes can be secreted by a wide range of mammalian cell types, including MSCs [20], immunocytes [21], neurons [22], cancerous cells [23], epithelial cells [24], osteocytes [25], and myocytes [26]. Meanwhile, exosomes can distribute in body fluids such as saliva [27], plasma [28, 29], lymph [30], urine [29], semen [31], and even breast milk [32]. Although exosomes can be separated from many sources, their morphology has been described as a cup-shaped appearance when visualized by transmission electron microscopy.

In terms of the isolation techniques of exosomes, a variety of novel techniques have been or are currently being developed, including ultracentrifugation-based isolation techniques, size-based techniques, precipitation techniques, immunoaffinity capture-based techniques, and some novel combination techniques [33]. These methods may be divided based on the differences in recovery and specificity, ranging from low to high in each dimension [34]. However, there is no one-size-fits-all model among the existing techniques, and complete isolation of exosomes from other components is unrealistic. Therefore, researchers must choose efficient, appropriate, and affordable techniques to separate exosomes. After isolation, exosomes can be stored at − 80 °C to maximize their functions [35]. However, several studies have shown that the biological functions of exosomes may be impaired even at − 80 °C, including morphological changes in exosomes [36] and degradation of exosomal RNA [37].

**Fig. 1:**
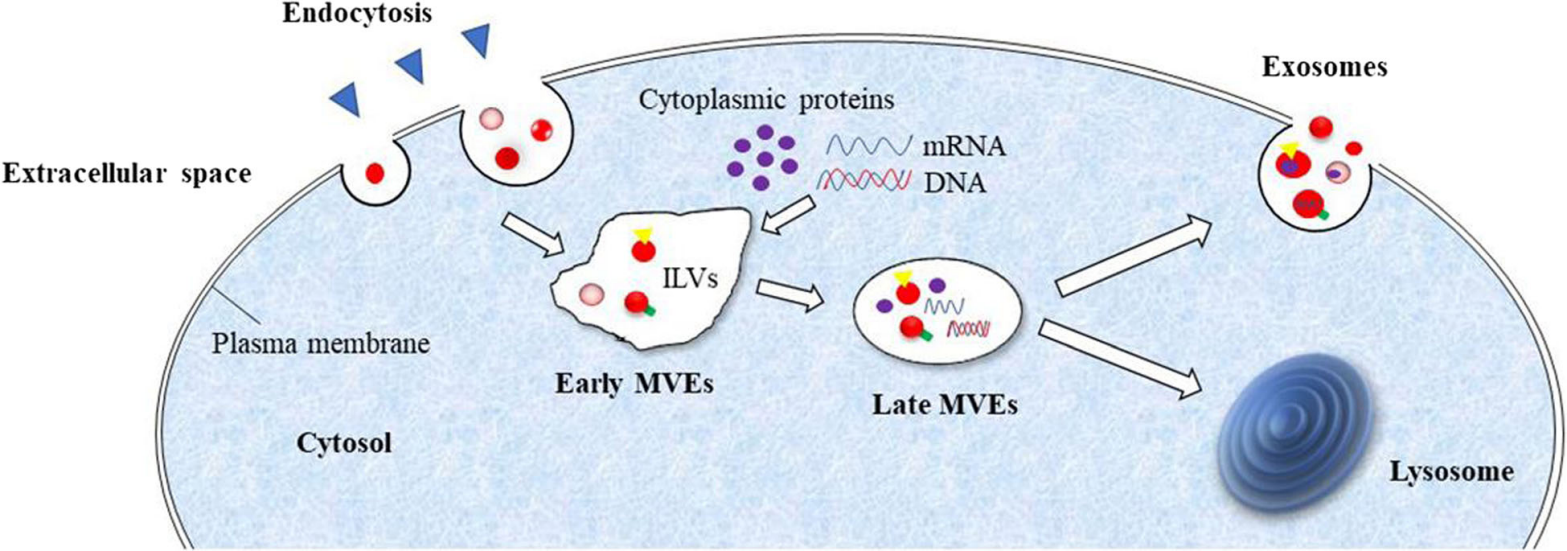
Pathways of exosome release. The process begins with the inward budding of the plasma membrane of endosomes, followed by transportation to early multivesicular endosomes (MVEs), which will undergo a series of changes into late MVEs with the accumulation of ILVs. After maturation, membranes of late MVEs generate and form vesicles that are 40–150 nm in size and contain various RNAs and proteins. Finally, the cargo of MVEs will be allocated to undergo two different pathways, delivered to lysosomes for degradation, or released into the extracellular milieu by fusing with the plasma membrane. The latter route gives rise to the production of exosomes

The biogenesis of exosomes is complicated and tightly regulated, involving multiple factors and signaling molecules such as tetraspanins, ceramide, and endosomal sorting complex responsible for transport (ESCRT) [38]. Furthermore, accumulating evidence indicates that the ESCRT pathway is involved in selecting and sorting proteins for intraluminal vesicles (ILVs), which are predestined to become exosomes [39, 40], and proteomic analyses of purified exosomes have identified subunits from ESCRT complexes and associated proteins, such as ALG2-interacting protein X (ALIX), ESCRT-II, Charged multivesicular body protein 2A (CHMP2A), CHMP4A/B/C, and vacuolar protein sorting 4 (VPS4) [41]. The simplified summary diagram of the most acceptable model of exosome formation and release is shown in Fig. 1.

Exosomes are encapsulated in a bilayer membrane that protects their genetic materials (DNA, mRNAs, miRNAs, pre-miRNAs, and other noncoding RNAs) and proteins transported to target cells [42–44]. Proteins enriched in exosomes include membrane transport proteins (GTPases and annexins), tetraspanins (CD63, CD81, CD82, and CD9), biogenesis-related proteins (ESCRT complex, ALIX, and tumor susceptibility gene (TSG) 101), and heat shock proteins (HSP60, 70, and 90) [45]. Furthermore, CD9, CD63, CD81, CD82, and abundant lipid rafts are generally recognized as characteristic biomarkers of exosomes [46, 47].

## Functions of adipose mesenchymal stem cell-derived exosomes

As described earlier, exosomes are involved in many biological processes due to their varied genetic materials and proteins. Zhang et al. [48] analyzed the miRNA profiles of ADSC-Exos and exosomes derived from adipose tissue (AT-Exos) and indicated that a total of 148 and 154 known miRNAs were identified in ADSC-Exos and AT-Exos, respectively. Furthermore, proteomic analysis of ADSC-Exos identified 1466 proteins that are involved in various cell functions [49]. Therefore, we will comprehensively describe the latest research progress on ADSC-Exos in different biological behaviors.

### Cell proliferation, migration, and apoptosis

**Fig. 2:**
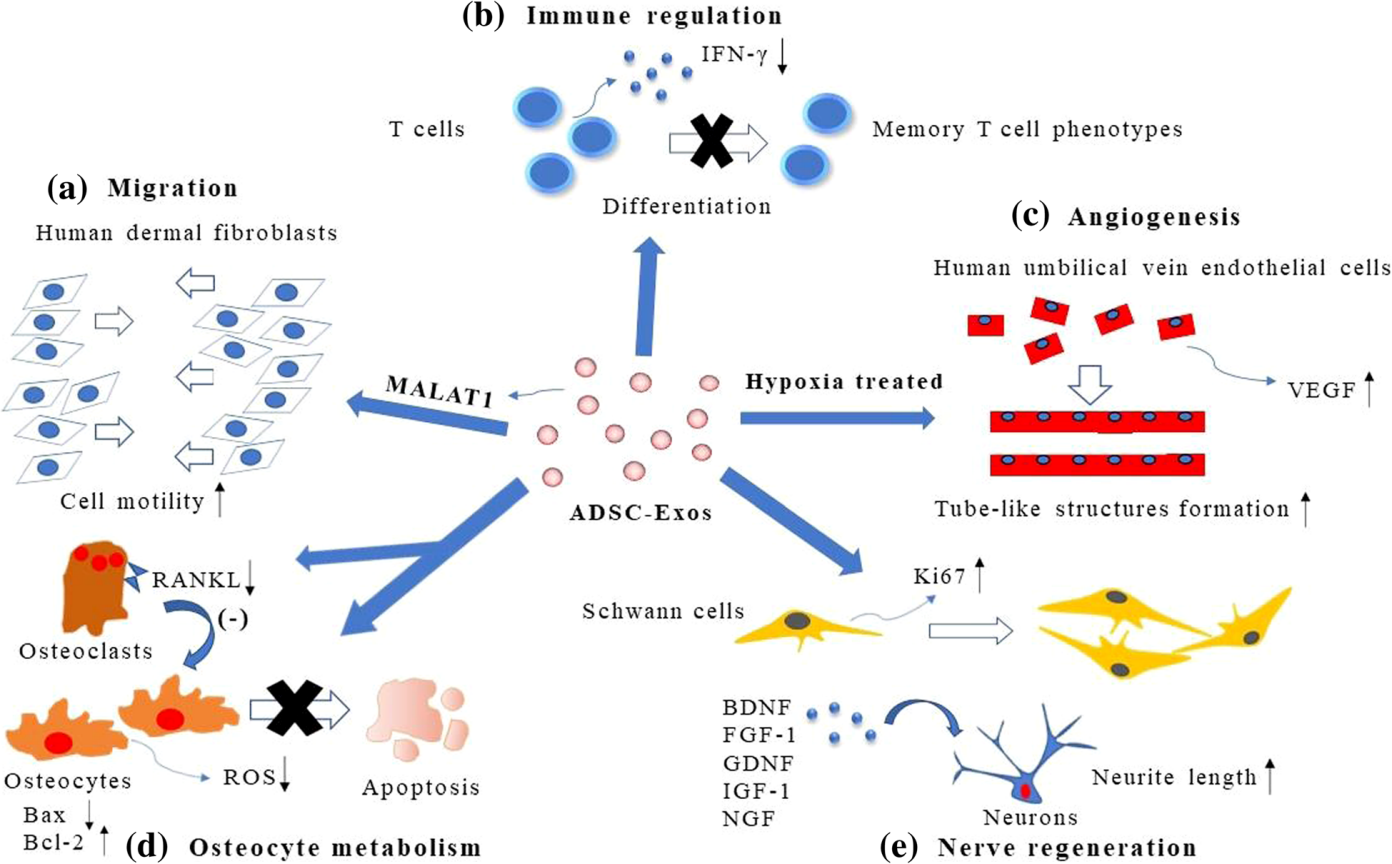
Functions of ADSC-Exos. (a) To enhance proliferation of HDFs by stimulating the expression of MALAT1, which is responsible for increasing cell motility. (b) To inhibit the differentiation of T cells into memory T cell phenotypes and the secretion of IFN-γ. (c) To promote angiogenesis of HUVECs by significantly increasing their tube-formation capability and VEGF secretion. (d) To protect osteocytes from apoptosis by suppressing the production of ROS, promoting the expression of the antiapoptotic gene Bcl-2 and inhibiting the expression of the proapoptotic gene Bax, and decreasing the expression of RANKL to antagonize osteoclastogenesis. (e) To promote neurite outgrowth by enhancing neurite lengths, along with the presence of neural growth factors (BDNF, FGF-1, GDNF, IGF-1, and NGF), and increasing the expression of cyclin Ki67 in Schwann cell nuclei, which is a marker of cell proliferation

Several studies have demonstrated the roles of ADSC-Exos in proliferation and migration via various mechanisms in different cell types, including vascular endothelial cells [50], tumor cells [51], epithelial cells [52], and fibroblasts [7] (Fig. 2 (a)). These vesicles promoted proliferation and migration of the breast cancer cell line MCF7 by activating the Wnt signaling pathway, which is engaged in tumor development and tissue homeostasis [53]. The protein β-catenin and Wnt target genes, such as Axin2 and Dickkopf-related protein 1 (Dkk1), were further confirmed to accumulate in this process. Moreover, ADSC-Exos could stimulate vessel formation and induce matrix metalloproteinase (MMP) secretion to increase invasion and migration of vascular endothelial cells [50]. Cooper et al. [54] found that ADSC-Exos could be easily internalized by human dermal fibroblasts (HDFs) and significantly promoted their migration, along with increased expression of long noncoding RNA (lncRNA) metastasis-associated lung adenocarcinoma transcript 1 (MALAT1), which is involved in the modulation of several molecular signaling pathways and affects proliferation, cell cycle, migration, angiogenesis, and tumorigenicity [55]. More importantly, the AKT and ERK signaling pathways, which are involved in the proliferation, migration, and tube formation of endothelial cells [56], have been demonstrated to be activated by ADSC-Exos via maximally elevating the phosphorylation of p-ERK1/2 and p-AKT [57]. Furthermore, ADSC-Exos can play an inhibitory role in cell apoptosis by transferring and regulating proteins and miRNAs. For example, Ma et al. found that ADSC-Exos promoted the proliferation and migration of HaCaT cells and inhibited their apoptosis after exposure to H_2_O_2_, while the decreased expression of the proapoptotic protein Bax and the increased expression of the antiapoptotic protein Bcl-2 both confirmed the validity of this conclusion [52]. Studies showed that exosomes collected from H_2_O_2_-treated MSCs contained higher levels of miR-21 than exosomes released from normal MSCs, which could protect against oxidative stress-triggered cell death [58]. In addition, in a skin flap transplantation model, noticeably fewer apoptotic cells were observed in the H_2_O_2_-treated ADSC-Exo group, resulting in higher skin flap survival after ischemia-reperfusion (I/R) injury [59]. Meanwhile, in another acute kidney IR injury model, apoptotic biomarkers (Bax/caspase-3/poly ADP-ribose polymerase) were significantly decreased by the combined use of ADSC-Exos and ADSCs [60]. In summary, ADSC-Exos have been shown to effectively promote the proliferation or migration of certain cell types (breast cancer cells, HaCaT cells, HDFs, and vascular endothelial cells) and markedly decrease the ratio of apoptotic cells. These findings undoubtedly indicate the crucial role of ADSC-Exos in tumor treatment and future therapeutic applications of regenerative medicine, such as chronic wound healing, severe burns, and post-operation scar formation.

### Regulation of immune and inflammatory responses

ADSCs have been demonstrated to possess an intrinsic capacity to alleviate inflammation and immune responses [61], which has been reported in certain cell types, such as natural killer T (NKT) cells [62], regulatory T cells [63], T cells [64], and dendritic cells [65]. Exosomes are thought to have similar immunomodulatory functions to cells by receiving their bioactive paracrine molecules [66] (Fig. 2 (b)). Concerning CD4+ and CD8+ T cells, ADSC-Exos have an inhibitory effect on their differentiation toward effector or memory cell phenotypes in vitro, which is mediated by anti-CD3/CD2/CD28 stimuli [67]. Furthermore, ADSC-Exos inhibit T cell activation by significantly reducing the secretion of interferon-gamma (IFN-γ), and they also lack MHC class II and costimulatory molecules, which indicates that these vesicles may have a direct inhibitory effect on activating T cells [67]. However, Zhao et al. [68] found that ADSC-Exos could be transferred into macrophages, increasing the mRNA levels of M2-related arginase-1 and IL (interleukin)-10. Moreover, ADSC-Exos induced macrophage polarization toward anti-inflammatory M2 phenotypes through the transactivation of arginase-1 by exosome-carried active signal transducer and activator of transcription 3 (STAT-3) and significantly inhibited macrophage inflammatory responses stimulated by lipopolysaccharide (LPS) plus IFN-γ [68]. However, the application of ADSC-Exos has been proven to ameliorate atopic dermatitis-like symptoms via regulating inflammatory responses and reducing the expression of inflammatory cytokines, such as IL-4, IL-23, IL31, and tumor necrosis factor-α (TNF-α), in a mouse model [69]. Furthermore, Chang et al. [61] found that healthy ADSC-Exos might be superior to apoptotic ADSC-Exos for improving survival and suppressing the inflammatory responses in mice after sepsis syndrome because the expression levels of inflammatory mediators (MMP-9, macrophage migration inhibitory factor, TNF-α, nuclear factor kappa-B (NF kappa B), IL-1β) were significantly higher in apoptotic ADSC-Exos than in healthy ADSC-Exos. However, there are still several debates and unanswered questions about the clinical use of ADSC-Exos, including their potential toxicity and specific dosages, which need to be further resolved.

### Promotion of angiogenesis

Angiogenesis is a complicated and highly regulated process that plays a crucial and critical role in different physiological events, including embryonic organ development, reproduction, tissue regeneration, and wound healing [70]. Recently, researchers have proven that exosomes derived from MSCs can promote angiogenesis [71] (Fig. 2 (c)). Notably, ADSC-Exos carried a subclass of miRNAs related to angiogenesis (miR-126, miR-130a, miR-132) [72, 73], which contribute to angiogenesis by increasing several growth factors in endothelial cells, such as vascular endothelial growth factor (VEGF), epidermal growth factor (EGF), and fibroblast growth factor (FGF) [74]. ADSC-Exos can also markedly enhance capillary network formation in human umbilical vein endothelial cells (HUVECs), while the hypoxic ADSC-Exo group had a better result [6]. Xue et al. [75] also found that hypoxia-treated ADSC-Exos enhanced angiogenesis through activating the PKA signaling pathway in HUVECs. Then, these cells enhanced the expression of VEGF and synergistically regulated the expression of the downstream proangiogenic genes Angpt1 and Flk1 while decreasing the angiogenesis inhibitory gene Vash. VEGF is a potent angiogenic signal protein that plays pivotal roles in regulating the formation of new blood vessels, such as induction of related gene expression, regulation of vascular permeability, and promotion of cell migration, proliferation, and survival, eventually promoting angiogenesis [76]. Moreover, a protein array showed angiogenesis-related protein enrichment of FGF, VEGF, EGF, and monocyte chemoattractant protein (MCP) levels in the hypoxia-treated ADSC-Exos compared to the normoxia-treated ADSC-Exos [76]. All these proteins have been demonstrated to promote vascular endothelial cell functions, which indicated that hypoxia-treated ADSC-Exos had a higher capacity to enhance angiogenesis. Nevertheless, Yang et al. [77] demonstrated that ADSC-Exos could promote the angiogenesis of brain microvascular endothelial cells after oxygen-glucose deprivation via elevating the expression of miR-181b-5p and then inhibiting the expression of its target transient receptor potential melastatin 7 (TRPM7), which represented a novel therapeutic approach for stroke recovery. These vehicles were proven to upregulate the protein expression of hypoxia-inducible factor 1α and VEGF and downregulate the protein expression of tissue inhibitor of metalloproteinase 3 [78]. Because hypoxia/normoxia-treated ADSC-Exos can affect angiogenesis to a certain extent, the application of these vesicles may play pivotal roles in fat grafting and some traumatic disease treatments in the future.

### Osteocyte metabolism

Emerging evidence has confirmed the vital paracrine effects of MSCs, especially secreted exosomes, for regulating osteocyte metabolism [79–81] (Fig. 2 (d)). Studies showed that the excessive production of reactive oxygen species (ROS), which provide contributions to activating cellular apoptosis through mechanisms involving mitochondrial pathways [82], was remarkably inhibited by ADSC-Exos in MLO-Y4 cells in hypoxia and serum deprivation (H/SD) conditions [80]. Moreover, ADSC-Exos antagonized H/SD-induced osteocyte apoptosis via upregulating the antiapoptotic protein Bcl-2 and repressing the proapoptotic protein Bax [80]. Meanwhile, data have shown that apoptotic osteocytes may exhibit enhancement of osteoclastogenesis by elevated receptor activator of the nuclear factor kappa-B ligand (RANKL) expression [83], while ADSC-Exos could efficiently revert this process by interacting with RANKL and then inhibit osteoclast activation [80]. However, ADSC-Exos could significantly inhibit the apoptosis of MLO-Y4 cells induced by a hypoxia-ischemia environment after they were treated with low-level laser irradiation [84]. In addition, Li et al. [79] found that the bone regeneration was enhanced by ADSC-Exos through its osteoinductive effects and ability to promote cell migration and homing when they were combined with polylactic acid-polyglycolic acid copolymer (PLGA) scaffolds, which were widely recognized as one kind of biocompatible and biodegradable biomaterial [85]. Anti-inflammatory effects on the osteocyte metabolism of osteoarthritis have also been demonstrated by ADSC-Exos, which were shown to enhance the production of the anti-inflammatory cytokine IL-10 [81]. Furthermore, these vesicles may significantly decrease the mitochondrial membrane changes and oxidative stress induced by IL-1β, which prolongs the downregulation of the inflammatory response [81]. Altogether, ADSC-Exos may play an effective and efficient role in inhibiting cellular senescence, correcting abnormal osteoblast metabolism, and exerting therapeutic potential in bone regeneration.

### Nerve regeneration

The role of exosomes derived from different cell types on neural regeneration has been studied (Fig. 2 (e)). For example, exosomes secreted from Schwann cells strongly enhanced neurite outgrowth [86] and provided an appropriate microenvironment for neuronal regeneration to occur [87]. However, a healthy nerve must be sacrificed to obtain Schwann cell exosomes, which will inevitably give rise to unwanted body injuries and ethical concerns. Therefore, studies have found an alternative approach utilizing ADSC-Exos to avoid these complications. Bucan et al. [88] found that ADSC-Exos could stimulate Schwann cell proliferation and increased expression of cyclin Ki67, indicating that exosomes could enhance neurite length of dorsal root ganglion (DRG) neurons. Moreover, the researchers demonstrated the presence of neural growth factors in the ADSC-Exos [88], such as brain-derived neurotrophic factor (BDNF), insulin-like growth factor-1 (IGF-1), nerve growth factor (NGF), FGF-1, and glial cell-derived neurotrophic factor (GDNF), which reveals their potential to be utilized as a therapeutic tool for nerve regeneration. In addition, low doses of ADSC-Exos increased the viability of and exerted antiapoptotic effects on neural cells by inhibiting the apoptotic cascade after those cells were exposed to oxidative damage with H_2_O_2_. In addition, exosomes could increase the process of remyelination and activate nestin-positive oligodendroglia precursors to exert their neuro-regeneration functions [89]. However, in a stress urinary incontinence (SUI) rat model, higher densities of striated muscle fibers and peripheral nerve fibers were also found in the urethra after ADSC-Exo treatment than those of the SUI group [49]. Furthermore, pigment epithelium-derived factor (PEDF), a 50-kDa secreted glycoprotein, has been shown to have a protective effect in cultured cortical neurons by inhibiting oxidative stress and apoptosis [90]. When ADSC-Exos were modified by PEDF, they strongly suppressed nerve cell apoptosis through a caspase-dependent (caspase-9 and caspase-3) pathway and activated autophagy by promoting the expression of autophagy-associated protein light chain 3 (LC3) [91]. In conclusion, in the future, the application of ADSC-Exos could potentially engender new approaches to nerve regeneration research. However, further exosome research still needs to be performed to uncover the underlying neurogenesis mechanisms and specific signaling molecules and pathways.

### Tumor growth

ADSCs play a pivotal role in the development of certain tumors, especially those with intimate and close connections, such as breast cancer and malignant melanoma. ADSCs promote migration, angiogenesis, or epithelial-mesenchymal transition of cancer cells in breast cancers [92] and malignant melanomas [93], while ADSC-Exos behave similarly in breast cancers. ADSC-Exos were demonstrated to promote migration and proliferation of MCF7 breast cancer cells, and some signaling pathways associated with tumor development were upregulated, of which the Wnt signaling pathway appeared to be the most prominent [51]. Therefore, there must be a link between ADSC-Exos and tumor cell migration. In another rat model of hepatocellular carcinoma (HCC), the ADSC-Exos showed suppression of tumorigenesis, as the exosome-treated group displayed significantly smaller tumors and volumes, a higher apparent diffusion coefficient, more NKT-cells, and low-grade HCC differentiation compared to the controls [94]. Currently, growing interest is focused on the utilization of exosomes as therapeutic biological delivery vehicles for miRNA and drug transfer [95]. Researchers have successfully used ADSC-Exos as a practical strategy to deliver miRNA-122 to enhance HCC chemosensitivity [96]. MiRNA-122 is highly expressed in normal liver tissue and has been shown to perform multiple functions in liver physiology and pathology. Overexpression of miR-122 was shown to inhibit cancer cell proliferation and metastasis and increase chemosensitivity of tumor cells [97]. Data showed that miR-122-transfected ADSC-Exos combined with sorafenib could efficiently increase the chemosensitivity of HCC cells by altering target gene expression, such as cyclin G1 (CCNG1), a disintegrin and metalloprotease 10 (ADAM10), and insulin-like growth factor receptor 1 (IGF1R), and then enhance cell apoptosis and cell cycle arrest in the G0/G1 population in vitro and in vivo [96]. These findings undoubtedly represent a novel exosome application to inhibit tumor formation. However, explorations of other tumor-related aspects of ADSC-Exos (including their roles in tumor angiogenesis, immunity, and the microenvironment) are lacking. Additional work is needed to enhance the ADSC-Exo applications for tumor treatment and confirm the optimal concentration for human use, as it will increase the feasibility and safety of ADSC-Exo therapy in clinical applications.

## Therapeutic potential of adipose mesenchymal stem cell-derived exosomes

The effects and therapeutic benefits of ADSC-Exos have been observed and confirmed in a wide range of diseases and are important in the development of future therapeutic applications, such as skin repair, regenerative engineering, and tumor applications (Table 1). Previously, researchers simply explored the basic functions of naturally derived exosomes and applied them to disease models with little modifications of their contents. However, many recent studies have revealed that some characteristics and contents of exosomes can be modified with other substances. In certain culture conditions, exosomes can serve as stable and effective vehicles loaded with specific proteins, lipids, and genetic materials, including mRNAs, miRNAs, other small noncoding RNAs, and DNA, thus serving as a promising tool for anticipated cargo delivery to targeted tissues or organs [98].

**Table 1:**
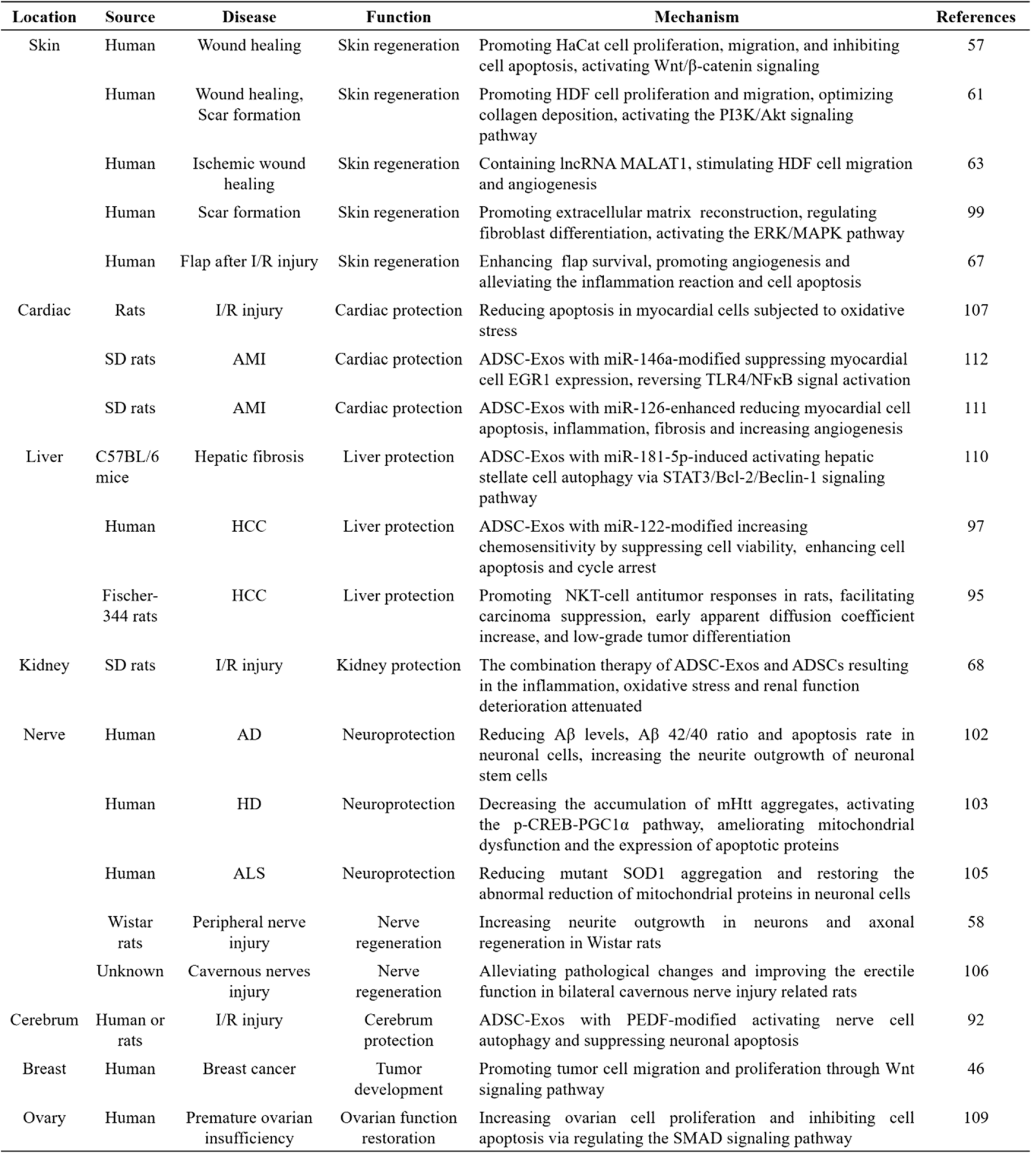
The mechanism and function of ADSC-Exos in different diseases

*HDF* human dermal fibroblasts, *lncRNA* long noncoding RNA, *MALAT1* metastasis-associated lung adenocarcinoma transcript 1, *I/R* ischemia-reperfusion, *SD rats* Sprague-Dawley rats, *AMI* acute myocardial infarction, *EGR1* early growth response factor 1, *HCC* hepatocellular carcinoma, *NKT-cell* natural killer T cell, *AD* Alzheimer’s disease, *Aβ* amyloid beta, *HD* Huntington’s disease, *mHtt* mutant Huntingtin, *ALS* amyotrophic lateral sclerosis, *SOD-1* superoxide dismutase 1, *PEDF* pigment epithelium-derived factor

As for ADSC-Exos, regardless of whether they have been modified, studies have already illustrated their essential functions in many biological and pathological processes. However, in terms of translational clinical research, a consensus in the doses of exosomes has not been reached, and the related studies are inadequate and limited. The optimal doses of exosomes seem to be varied according to different models. For example, in a wound healing model, the optimal concentration of exosomes was confirmed to be 50 μg/ml in vitro [7]. However, a concentration of 40 μg/ml ADSC-Exos has been shown to effectively promote adipogenic differentiation in vitro [48]. Furthermore, in a diabetic erectile dysfunction rat model, the 100 μg exosome treatment group was shown to have stronger therapeutic effects than the 10 μg group [72]. Since the doses of exosomes are closely related to future clinical practice and biological safety, it is crucial and critical for us to set the concentration gradient in our exosome studies and search for the optimal concentrations in diverse fields.

Next, the therapeutic potential of ADSC-Exos will be explicitly described in many clinical aspects, including the progress and limitations of existing research.

### Cutaneous healing and regeneration

Cutaneous healing and regeneration are complicated processes and require well-orchestrated integration of multiple biological and molecular events, including cell migration, differentiation, proliferation, apoptosis, and extracellular matrix deposition [99]. At present, the key problems are concentrated on delayed cutaneous healing and excessive scar formation, while ADSC-Exos are proven to solve both of these issues. ADSC-Exos can be internalized by fibroblasts and promote their proliferation and migration and increase collagen type I and III deposition via the PI3K/Akt signaling pathway [7]. Moreover, ADSC-Exos could promote proliferation and migration and inhibit apoptosis of HaCaT cells via Wnt/β-catenin signaling [52]. Another study revealed that ADSC-Exos contained MALAT1, which has the potential to stimulate the migration and angiogenesis of HDFs [54]. These findings all indicated the active role of ADSC-Exos in accelerating cutaneous wound healing.

Regarding scar formation, markedly enhanced re-epithelialization and narrower scar areas were also observed in the wounds of mice treated with ADSC-Exos [7]. Moreover, ADSC-Exos could promote extracellular matrix reconstruction in cutaneous wound regeneration by regulating the proportions of collagen type III: type I, transforming growth factor beta 3 (TGF-β3): TGF-β1 and MMP3: tissue inhibitor of metalloproteinases 1 (TIMP1), and preventing the differentiation of fibroblasts into myofibroblasts to attenuate scar formation [100]. In addition, with regard to treating a refractory wound, one of the most important strategies is related to flap transplantation. Studies have shown that the use of ADSC-Exos can reduce flap necrosis, promote neovascularization, and alleviate the inflammatory reaction and apoptosis in the skin flap after I/R injury [59]. In summary, the results from all of these related studies suggest that ADSC-Exos could be a promising cell-free therapeutic strategy for the treatment of cutaneous healing and regeneration. However, more details about the molecular mechanism must be elucidated, and clinical trials of ADSC-Exos must be conducted.

### Neurodegenerative disorders

In many neurodegenerative diseases, ADSC-Exos have been shown to play a pivotal role in neuroprotection and neuroregeneration. Alzheimer’s disease (AD) is a type of dementia characterized by cognitive deficits and pathologically by the accumulation of amyloid beta (Aβ) plaques, together with progressive neuronal death in the hippocampus and the cerebral cortex [101]. However, the increased Aβ levels and the Aβ42/40 ratio of AD neuronal cells could be decreased by ADSC-Exo treatment. The apoptosis rate of AD neuronal cells was significantly reduced by ADSC-Exo because increased cell survival and decreased cell death were observed. These vesicles also augmented neurite outgrowth of neuronal stem cells [102]. Taken together, these findings indicated the potential of ADSC-Exos as a therapeutic tool for treating AD. Huntington’s disease (HD) is a type of hereditary neurodegenerative disorder caused by the aggregation of mutant Huntingtin (mHtt). Lee et al. [103] found that ADSC-Exos decreased the accumulation of mHtt aggregates, activated the p-CREB-PGC1α pathway, and ameliorated mitochondrial dysfunction and the expression of apoptotic proteins in an in vitro HD model. This finding highlighted the possible therapeutic function of ADSC-Exos in treating HD. In addition, amyotrophic lateral sclerosis (ALS), which is a degenerative disorder characterized by pathologic hallmark mutant superoxide dismutase 1 activation and aggregation [104], could be treated by ADSC-Exos. This treatment decreased mutant superoxide dismutase 1 (SOD-1) aggregation in G93A neuronal cells and normalized cellular phenotypes of ALS, thus restoring the abnormal reduction of mitochondrial proteins including p-CREB and PGC-1α [105].

Moreover, in peripheral nerve injury models, including sciatic nerve injury and cavernous nerve injury, ADSC-Exos have been proven to be helpful for nerve regeneration. These vesicles increased neurite outgrowth of DRG neurons in vitro and enhanced axonal regeneration after sciatic nerve injury in vivo [88]. Furthermore, in a rat model of bilateral cavernous nerve injury, ADSC-Exo treatment could significantly alleviate pathological changes, including the distortion of normal neural anatomy, smooth muscle atrophy, and collagen deposition, and improve their erectile function [106]. Although the use of ADSC-Exos has been reported to be an effective and safe treatment for some certain central nervous system and peripheral nervous system diseases, exosome-related studies on many other neurological diseases are lacking, and further research is needed.

### Ischemia-reperfusion

Data have shown that the occurrence of IR in any tissue/organ rapidly results in progressive inflammatory cell recruitment, endoplasmic reticulum stress, post-ischemic capillary no-reflow, and superfluous production of reactive oxidative stress, resulting in organ failure or serious irreversible damage to the human body [107]. Currently, ADSC-Exos, as a novel cell-free therapy, are increasingly considered a new tool for the treatment of IR injury. For example, ADSCs-Exos can protect myocardium from I/R injury through activating the Wnt/β-catenin signaling pathway by exerting antiapoptotic and prosurvival effects on cardiomyocytes [108]. Furthermore, while oxidative stress was crucial to the development of ischemia-reperfusion injury, the application of ADSC-Exos could protect myocardial cells by reducing apoptosis subject to oxidative stress in vitro [109]. Another essential finding is that ADSC-Exo therapy protects the kidney from IR injury. The combination therapy of ADSC-Exos and ADSCs is better than using either one alone to protect the kidney from acute IR damage, resulting in strong attenuation of inflammation, oxidative stress, and renal function deterioration. Intriguingly, ADSC-Exos could also increase angiogenesis and blood flow, conserving the function and structure of the kidney [60]. In addition, exosomes can serve as therapeutic and safe vehicles in which genetic material or other helpful contents can be transferred or stimulated. Therefore, as we mentioned before, studies have shown that PEDF-modified ADSC-Exos ameliorated cerebral I/R injury by activating autophagy and suppressing neuronal apoptosis [91]. In the skin flap after IR damage, ADSC-Exos could promote flap survival, facilitate neovascularization, and alleviate inflammatory reactions and apoptosis after being stimulated by hydrogen peroxide [59]. In conclusion, although there are few effective clinical treatments for ischemia-reperfusion injury at present, cell-free therapies such as ADSC-Exos may be a valuable tool in enhancing recovery after I/R injury.

### Parenchymal organ diseases

As mentioned above, ADSC-Exos could promote or restrain cancer development in different microenvironments. For example, in a breast cancer cell model, ADSC-Exos promoted migration through the Wnt signaling pathway [51]. However, in an HCC rat model, these vesicles facilitated NKT cell antitumor responses, thereby playing an inhibitory role in HCC [94]. Furthermore, when transfected with a miR-122 expression plasmid, ADSC-Exos could increase the sensitivity of HCC to chemotherapeutic agents in vitro and in vivo [96]. According to this dual character, researchers need to find a way to balance and use exosomes as an effective treatment for malignant tumors.

ADSC-Exos could restore ovarian function via increasing the proliferation rate and inhibiting the apoptosis rate of the ovarian granule cells by regulating the SMAD signaling pathway, which were confirmed to improve the ovarian function in premature ovarian insufficiency disease [110]. Currently, an increasing number of studies have concentrated on the use of miRNA-transfected ADSC-Exos for therapeutic delivery to target diseases. For example, in liver fibrosis, the miR-181-5p-modified ADSC-Exos were shown to have a therapeutic effect. In a rat model of hepatic fibrosis and mouse hepatic stellate cells, ADSC-Exos could activate cell autophagy and reduce TGF-β1-induced hepatic fibrosis via suppressing the STAT3/Bcl-2/Beclin1 pathway [111]. In addition, in cardiovascular diseases, the miR-126-enriched expression of ADSC-Exos could protect myocardial cells from apoptosis, inflammation, and fibrosis and increase their angiogenesis, thus preventing acute myocardial infarction (AMI) [112]. In addition, in another AMI rat model and hypoxic-induced H9c2 cell model, the miR-146a-modified ADSC-Exos were shown to effectively attenuate AMI-induced myocardial damage through downregulation of early growth response factor 1 and then reversed AMI or hypoxia-induced TLR4/NFκB signal activation [113]. In brief, in a comparison with treatment with only ADSC-Exos, strategies allowing exosomes to transport miRNAs to treat parenchymal organ diseases showed stronger potential. However, to date, the organ diseases that can be effectively treated by ADSC-Exos are limited. More studies are needed to explore this promising field.

### Obesity

Inflammation originating from adipose tissue is considered to be one of the main causes of the development of insulin resistance and type 2 diabetes in obese individuals [114]. Studies have shown that ADSCs play pivotal roles in obesity-associated inflammation and metabolic disorders. Soluble factors secreted from ADSCs, such as exosomes, were shown to affect obesity and diabetes. ADSC-Exos were demonstrated to dominate the polarization of anti-inflammation (M2) macrophage phenotypes, thus remodeling the immune homeostasis in white adipose tissue (WAT) [115]. Exosomes could activate M2 macrophage polarization and inflammation reduction in the WAT of obese mice [68]. Notably, ADSC-Exos-induced M2 macrophages could express high levels of tyrosine hydroxylase responsible for catecholamine release [68], which activated the expression of brown adipose tissue–specific uncoupling protein 1 in WAT and promoted fat burning to dissipate extra energy. Collectively, these findings indicate indispensable exosome-mediated crosstalk between ADSCs and macrophages, shedding light on the potential of an exosome-based therapeutic approach to control obesity. Nevertheless, currently, ADSC-Exo-related studies on obesity are rare, and more investigations are still needed to expand our understanding of exosome functions in obesity.

## Limited therapeutic effects of ADSC-Exos

Exosomes, especially ADSC-Exos, have gradually become multipotent and multifunctional frontiers in contemporary medicine. However, there are still some limitations of ADSC-Exos in certain areas that need to be mentioned. For example, our recent studies have provided a novel regulatory mechanism of ADSC adipogenic differentiation from the perspective of lncRNA-miRNA-mRNA (H19/miR-30a/C8orf4 axis) regulation [116], which is important for obesity-related disorders and adipose tissue regeneration. However, the function and precise molecular mechanism in ADSC-Exo adipogenic differentiation are unclear. Studies have even shown that only AT-Exos could induce adipogenesis, while ADSC-Exos had little effect [48]. The reason could be the complex composition of adipose tissue, resulting in the adipogenic miRNAs and proteins being relatively enriched. Moreover, given the multiple types of human tumors, few studies of ADSC-Exos have demonstrated therapeutic effects in tumors. The potential reasons may be primarily due to the inherited characteristics of ADSCs, which are mainly associated with breast cancer and other adipose-related tumors, and the few explorations of ADSC-Exos in tumors to date.

## Conclusions and prospects

Exosomes, which are extensively involved in cell-to-cell communication and cell signaling transportation, are capable of altering a wide range of biological processes, such as proliferation, migration, and apoptosis. Exosomes derived from ADSCs have gradually attracted increased interest and shown application prospects because of their wide range of sources and easy accessibility. Corresponding studies on these vesicles have already shown their wide range of clinical therapeutic applications, including tissue regeneration, immune responses, tumors, and many other essential fields. Collectively, ADSC-Exos could serve as an available and effective candidate in cell-free therapeutic medicine and may become a suitable alternative to single-applied ADSCs. Relevant studies will pave the way for industrial production of ADSC-Exos.

In the future, however, many questions concerning the application of exosomes remain to be solved. For example, there are few studies published on ADSC-Exos at present, many of which were short-term studies that did not show a long-lasting therapeutic effect of ADSC-Exos. The specific therapeutic safe doses of ADSC-Exos for human use are still unknown. Hence, the next step should be investigating the biosafety range of ADSC-Exos. Long-duration and large-sample in vivo studies are definitely needed to broaden our knowledge of exosomes.

## Data Availability

Data sharing is not applicable to this article as no datasets were generated or analyzed during the current study.

## Abbreviations

AD: Alzheimer’s disease
ADAM10: A disintegrin and metalloprotease 10
ADSC: Adipose-derived stem cell
ADSC-Exos: Exosomes derived from adipose-derived stem cells
ALIX: ALG2-interacting protein X
ALS: Amyotrophic lateral sclerosis
AMI: Acute myocardial infarction
AT-Exos: Exosomes derived from adipose tissue
Aβ: Amyloid beta
BDNF: Brain-derived neurotrophic factor
CCL5: C-C motif chemokine ligand 5
CCNG1: Cyclin G1
CHMP2A: Charged multivesicular body protein 2A
Dkk1: Dickkopf-related protein 1
DRG: Dorsal root ganglion
EGF: Epidermal growth factor
EGR1: Early growth response factor 1
ESCRT: Endosomal sorting complex responsible for transport
EVs: Extracellular vesicles
FGF-1: Fibroblast growth factor-1
GDNF: Glial cell-derived neurotrophic factor
H/SD: Hypoxia and serum deprivation
HCC: Hepatocellular carcinoma
HD: Huntington’s disease
HDF: Human dermal fibroblast
HSP: Heat shock protein
HUVECs: Human umbilical vein endothelial cells
I/R: Ischemia-reperfusion
IFN-γ: Interferon-gamma
IGF-1: Insulin-like growth factor-1
IGF1R: Insulin-like growth factor receptor 1
IL: Interleukin
ILVs: Intraluminal vesicles
LC3: Light chain 3
lncRNA: Long noncoding RNA
LPS: Lipopolysaccharide
MALAT1: Metastasis-associated lung adenocarcinoma transcript 1
MCP: Monocyte chemoattractant protein
mHtt: Mutant Huntingtin
miRNAs: MicroRNAs
MSCs: Mesenchymal stem cells
NF kappa B: Nuclear factor kappa-B
NGF: Nerve growth factor
NKT-cell: Natural killer T cell
NPM1: Nucleophosmin 1
NUP62: Nucleoporin 62
PDCD4: Programmed cell death 4
PEDF: Pigment epithelium-derived factor
PI3K: Phosphoinositide 3-kinase
PLGA: Polylactic acid-polyglycolic acid copolymer
RANKL: Receptor activator of the nuclear factor kappa-B ligand
ROS: Reactive oxygen species
SD rats: Sprague-Dawley rats
SOD-1: Superoxide dismutase 1
STAT-3: Signal transducer and activator of transcription 3
SUI: Stress urinary incontinence
TGF-β: Transforming growth factor beta
TIMP1: Tissue inhibitor of metalloproteinases 1
TNF-α: Tumor necrosis factor alpha
TRPM7: Transient receptor potential melastatin 7
TSG101: Tumor susceptibility gene
VEGF: Vascular endothelial growth factor
VPS4: Vacuolar protein sorting 4
WAT: White adipose tissue
